# Mirror Therapy Versus Motor Imagery in Stroke Neurorehabilitation: A Systematic Review with Comparative Narrative Synthesis

**DOI:** 10.3390/life16020306

**Published:** 2026-02-10

**Authors:** Luis Polo-Ferrero, Javier Torres-Alonso, Juan Luis Sánchez-González, Sara Hernández-Rubia, Arturo Dávila-Marcos, María Agudo Juan, Javier Oltra-Cucarella, Rubén Pérez-Elvira

**Affiliations:** 1 Department of Nursing and Physiotherapy, Universidad de Salamanca, 37007 Salamanca, Spain; javiertorres@usal.es (J.T.-A.); sarahernandezrubia@usal.es (S.H.-R.); turitodavila@usal.es (A.D.-M.); 2 Instituto de Investigación Biomédica de Salamanca (IBSAL), 37007 Salamanca, Spain; juanluissanchez@usal.es; 3 Department of Medicine, Universidad de Salamanca, 37007 Salamanca, Spain; 4 Laboratory of Neuripsychophysiology, NEPSA Rehabilitación Neurológica, 37003 Salamanca, Spain; mjagudojuan@gmail.com (M.A.J.); rperezel@upsa.es (R.P.-E.); 5 Department of Health Psychology, Universidad Miguel Hernández de Elche, 03202 Alicante, Spain; 6 Department of Psychobiology, Pontifical University of Salamanca, 37002 Salamanca, Spain

**Keywords:** stroke rehabilitation, mental practice, visual feedback, motor recovery, functional outcomes

## Abstract

**Background:** Motor imagery (MI) and mirror therapy (MT) are widely used neurorehabilitation strategies to enhance motor recovery after stroke and are commonly applied as adjuncts to conventional rehabilitation therapy (CRT). However, direct comparative evidence between these interventions remains limited. This systematic review compared the effects of MI and MT on motor function, functional performance, spasticity, and gait-related outcomes in adults after stroke. **Methods:** A systematic comparative review with narrative synthesis was conducted following PRISMA guidelines and registered in PROSPERO (CRD420251274308). PubMed, Cochrane Library, CINAHL, Scopus, Web of Science, and ScienceDirect were searched up to July 2025. Clinical trials directly comparing MI and MT in adults with stroke were included. Methodological quality was assessed using the PEDro scale, and risk of bias was evaluated with the Cochrane RoB 2 tool. **Results:** Six clinical trials involving 206 participants were included. Both MI and MT were associated with significant pre–post improvements across motor function, functional performance, spasticity, and gait-related outcomes. Between-group comparisons yielded heterogeneous findings, with no consistent evidence supporting the superiority of either intervention. Isolated advantages of MI were reported for specific upper-limb subdomains, but these effects were not consistently replicated. Overall methodological quality ranged from low to moderate, and all included studies were judged to be at high risk of bias according to the RoB 2 tool. **Conclusions:** MI and MT appear to provide comparable benefits for motor and functional recovery after stroke when used as adjuncts to CRT. Current evidence does not support the preferential use of one intervention, highlighting the need for well-designed trials with improved methodological rigor.

## 1. Introduction

Stroke remains one of the leading causes of long-term motor disability in adults worldwide, frequently resulting in persistent impairments of upper and lower limb function [1,2]. Despite substantial advances in acute management and conventional rehabilitation (CRT), a large proportion of stroke survivors experience incomplete functional recovery, with residual motor deficits that significantly compromise independence and quality of life [3]. These limitations have driven ongoing efforts to identify complementary rehabilitation strategies capable of enhancing motor recovery beyond what can be achieved through physical practice alone [4].

Contemporary neurorehabilitation is increasingly grounded in the principles of neuroplasticity, emphasizing task-specific training, repetition, and the activation of motor-related neural networks to promote cortical reorganization [5]. Within this framework, interventions that engage the motor system without requiring overt movement have gained particular relevance, especially for patients with severe motor impairment or limited capacity for active practice [6,7]. Among these approaches, motor imagery (MI) and mirror therapy (MT) have emerged as widely used, non-invasive techniques designed to stimulate motor networks through mental simulation and visual feedback, respectively [6,7,8].

MI is defined as the conscious mental rehearsal of motor actions without their physical execution [9,10]. Neuroimaging and neurophysiological studies have shown that MI activates cortical and subcortical regions largely overlapping with those involved in actual movement execution [11]. On this basis, MI has been incorporated into stroke rehabilitation as an adjunct to CRT, with several reviews reporting beneficial effects on motor and functional outcomes [12,13,14,15]. However, more recent meta-analyses applying rigorous methods and controlling for publication bias have questioned these findings, indicating that the observed effects are not robust and often disappear after bias correction [16,17]. Thus, despite its low physical demand and theoretical appeal, the clinical effectiveness of MI as an adjunct to CRT remains uncertain.

MT is based on mirror-induced visual feedback that creates the illusion of movement of the affected limb, engaging mirror neuron-related mechanisms and modulating sensorimotor cortical excitability [18]. Recent systematic reviews and meta-analyses show that MT improves upper-limb motor function after stroke, with greater effects reported for unilateral protocols and for virtual reality-based delivery [19,20,21,22]. Evidence also supports beneficial effects on lower-limb motor recovery, balance, and gait outcomes [23,24]. Clinically, MT is easy to implement and low-cost, although its effectiveness may be limited in patients with visuospatial deficits, including unilateral neglect [25].

Although both MI and MT have been explored as adjuncts to CRT, the consistency of the evidence supporting their clinical effectiveness differs. While MT shows more stable benefits across several motor and functional outcomes, evidence for MI remains heterogeneous and uncertain, particularly when methodological rigor and publication bias are considered. Direct comparisons between MI and MT are scarce, and, to the best of our knowledge, no previous systematic review has specifically synthesized and contrasted the effects of these two interventions across multiple motor and functional domains. Moreover, the extent to which patient-related characteristics may modulate the comparative effects of MI and MT remains poorly defined, particularly in the context of direct head-to-head comparisons. Despite their widespread use as adjuncts to conventional rehabilitation, it remains unclear whether MI and MT differ in their effects when directly compared, as most available evidence derives from separate, non-comparative trials. Therefore, this systematic narrative review specifically aims to test whether differences exist between motor imagery and mirror therapy with respect to motor function, functional performance, spasticity, and gait-related outcomes in adults after stroke, based exclusively on studies providing direct head-to-head comparisons between both interventions.

## 2. Materials and Methods

### 2.1. Review Protocol and Registration

This systematic review was conducted in accordance with the Preferred Reporting Items for Systematic Reviews and Meta-Analyses (PRISMA) guidelines [26]. To enhance methodological transparency and prevent duplication of research efforts, the review protocol was prospectively registered in the International Prospective Register of Systematic Reviews (PROSPERO) (registration number: CRD420251274308).

### 2.2. Study Eligibility and Inclusion Criteria

Study eligibility was guided by the PICOS framework (Population, Intervention, Comparison, Outcomes, and Study design), which guided the selection of the studies ultimately included in the review [27]. 

The review included studies conducted in adults with stroke presenting upper- and/or lower-limb motor impairment, irrespective of sex, stroke etiology, lesion location, or post-stroke phase (acute, subacute, or chronic). Studies enrolling only children or adolescents were excluded, as were those including participants with severe cognitive impairment, apraxia, neglect, or global aphasia when these conditions prevented active engagement with the interventions.

Eligible interventions consisted of MI-based rehabilitation, defined as the guided mental rehearsal of motor tasks with or without audiovisual support, and delivered either alone or in combination with CRT, provided that MI represented a clearly identifiable component of the intervention. Studies were required to include MT as the comparator intervention, implemented through mirror-induced visual feedback targeting the upper or lower extremity and delivered alone or alongside CRT.

To be eligible, studies had to report at least one motor or functional outcome, including measures of motor function, functional performance, spasticity, or gait-related parameters assessed with validated instruments. 

Only comparative experimental studies, including randomized controlled trials (RCTs) and non-RCTs directly comparing MI and MT, were considered. Observational studies without a comparator group, case reports, conference abstracts, protocols, and secondary research were excluded.

### 2.3. Search Strategy

A structured literature search was conducted to retrieve studies relevant to the objectives of this review. Searches were performed in PubMed, the Cochrane Library, CINAHL, Scopus, Web of Science, and ScienceDirect, covering the period from database inception to July 2025. For each database, a specific search strategy was developed, combining controlled vocabulary terms (e.g., MeSH) and free-text keywords, which were linked using Boolean operators to optimize retrieval of pertinent records.

Only articles published in English were considered eligible, which may have resulted in the exclusion of potentially relevant studies published in other languages. No limits were imposed on the year of publication. Gray literature was explored through manual screening of conference proceedings and trial registries, as well as by reviewing the reference lists of relevant reviews and all included studies. In addition, targeted searches were conducted to identify unpublished or ongoing studies when sufficient methodological information was available. To minimize the risk of missing relevant studies, the reference lists of all included articles were examined manually. Detailed search strategies for each database are available in Table S1, allowing full reproducibility of the search process.

### 2.4. Study Selection

All records retrieved from the electronic searches were uploaded to Rayyan software (https://www.rayyan.ai/, accessed on 7 February 2026), for reference management and screening. Duplicate entries were identified and removed prior to the selection process. Two reviewers (L.P.-F. and J.T.-A.) independently assessed titles and abstracts to identify potentially eligible studies in accordance with the predefined inclusion criteria. Articles considered relevant at this stage underwent full-text evaluation to determine final eligibility. Disagreements between reviewers were resolved through discussion, and when consensus was not achieved, a third reviewer (J.L.-S.-G.) was consulted. To ensure comprehensive study identification, the reference lists of all included articles were also screened manually. When necessary, study authors were contacted to clarify methodological aspects or to obtain missing information.

### 2.5. Data Extraction

All relevant data were extracted from each eligible study by a single reviewer using a predefined data extraction form. Following extraction, the collected information was reviewed to verify completeness and logical coherence, and any uncertainties or ambiguities were resolved through discussion within the research team.

Extracted data included bibliographic information (authors, year of publication, country, and study design), participant characteristics (sample size, age, sex distribution, stroke phase, stroke etiology when reported, and cognitive status), and detailed characteristics of the MI and MT interventions. Intervention-related data comprised the targeted limb, task type, session duration, intervention frequency, total intervention length, delivery modality, and integration with CRT. Information regarding comparator interventions and outcome domains—motor function, functional performance, spasticity, and gait—was also collected, together with the assessment instruments used and the timing of outcome evaluations.

Outcome data were examined qualitatively, with emphasis on the consistency and direction of reported effects. Numerical values, when available, were used descriptively to support interpretation, and no quantitative synthesis was performed. 

### 2.6. Narrative Data Synthesis and Analysis

Due to the limited number of included studies and substantial heterogeneity across trials in terms of intervention characteristics, targeted limbs, and lack of full consistency in the outcome variables assessed, a quantitative synthesis was not appropriate. Therefore, a narrative qualitative synthesis was conducted.

Study findings were organized and summarized descriptively according to predefined outcome domains (motor function, functional performance, spasticity, and gait-related outcomes), with emphasis on the direction, consistency, and presence or absence of between-group differences between MI and MT. The narrative synthesis followed a structured, outcome-domain-based approach, in which findings were first grouped by intervention comparison and then qualitatively contrasted within each domain. When available, means and standard deviations were calculated from reported descriptive data to support qualitative comparisons across studies. No statistical pooling or assessment of publication bias was performed, given the small number of studies and the heterogeneity of the analyzed variables. The development of the narrative synthesis is explicitly presented in Section 3.3 (Summary of Results), where findings are reported and contrasted domain by domain.

### 2.7. Methodological Quality and Bias Assessment

The risk of bias of the included studies was evaluated using the Cochrane Risk of Bias tool, version 2 (RoB 2) for RCTs [28]. Each study was assessed across the predefined domains of the tool, including the randomization process, deviations from intended interventions, missing outcome data, measurement of outcomes, and selection of reported results. Based on the judgments across these domains, an overall risk-of-bias judgment was assigned to each study following the RoB 2 guidance. Assessments were performed independently by two reviewers. Inter-rater reliability was evaluated using Cohen’s kappa [29], calculated for each domain and for the overall judgment, and interpreted according to established thresholds [30]. Disagreements were resolved through discussion and, when necessary, consultation with a third reviewer to reach consensus.

In addition, the methodological quality of the included trials was appraised using the Physiotherapy Evidence Database (PEDro) scale [31]. This scale consists of 11 items assessing key methodological aspects related to external validity, internal validity, and statistical reporting. Item 1 evaluates eligibility criteria and is not included in the total score, which is calculated by summing items 2 to 11, yielding a maximum score of 10 points. In accordance with established conventions, PEDro scores were interpreted as follows: scores <4 indicated poor methodological quality, scores of 4–5 fair quality, scores of 6–8 good quality, and scores of 9–10 excellent quality [32].

## 3. Results

### 3.1. Overview of Study Identification and Eligibility Assessment

A comprehensive search across six electronic databases resulted in 4074 potentially relevant references (PubMed, n = 581; Cochrane Library, n = 507; ScienceDirect, n = 756; Scopus, n = 846; Web of Science, n = 1113; and CINAHL, n = 271). After consolidation of the search outputs and elimination of duplicate entries (n = 1684), 2390 records were subjected to an initial screening process.

During title and abstract screening, 2187 publications were excluded because they did not align with the established eligibility framework. Full texts were subsequently examined for 203 studies, of which 197 were excluded following in-depth assessment. These exclusions were primarily attributable to records identified solely as trial registrations (n = 94), methodological or population mismatches with the inclusion criteria (n = 90), abstract-only publications (n = 11), or the absence of accessible full-text versions (n = 2).

**Figure 1:**
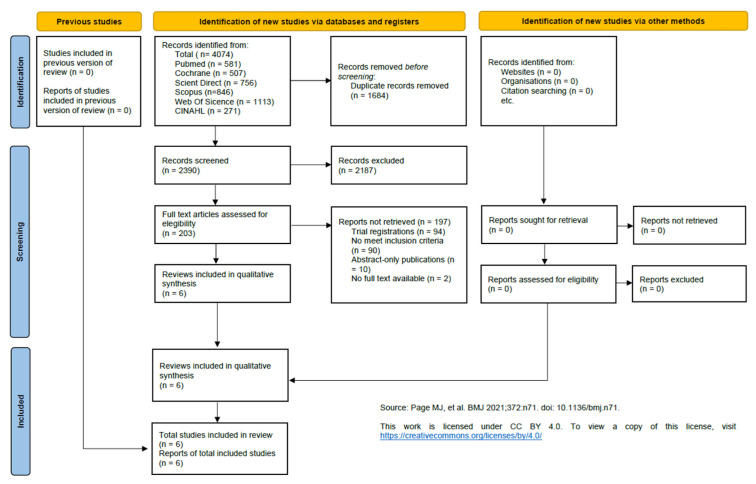
PRISMA 2020 flow diagram illustrating the study selection process [26].

As a result, six RCTs met all inclusion requirements and were retained for qualitative synthesis. The selection pathway from identification to final inclusion is summarized in the PRISMA flow diagram (Figure 1). 

### 3.2. Characteristics of the Included Studies

The main characteristics of the included studies are summarized in Table 1. The following sections describe key aspects of study design, participant characteristics, interventions, and outcome measures relevant to the comparative analysis of MI and MT.

#### 3.2.1. Study Design and Sample Size

Of the six included studies, five were RCTs with parallel two-arm designs comparing MI and MT. The remaining study employed a comparative experimental design with four intervention arms, in which MI and MT were analyzed according to cognitive status (moderate and severe impairment); however, the method of randomization was not clearly described [33]. Across all studies, a total of 206 participants were included, with 103 allocated to MI interventions and 103 to MT interventions. All studies were characterized by small sample sizes, ranging from 24 to 62 participants. Group sizes varied between approximately 6 and 31 participants, with the most common group size being 15 participants per group [34,35,36,37], resulting in a mean group size of 14.4 ± 8.0 participants. The main characteristics of the included studies are summarized in Table 1. The following sections describe key aspects of study design, participant characteristics, interventions, and outcome measures relevant to the comparative analysis of motor imagery and mirror therapy.

#### 3.2.2. Participant Demographics

Participants were predominantly middle-aged to older adults, with mean ages generally between 50 and 65 years. One study reported a broader age range extending up to 80 years [37]. Two studies reported age ranges rather than mean values (50–65 years [34]; 30–80 years [38]), while one study did not report mean age values [35]. Sex distribution was not reported in half of the included studies [33,34,38]. Among studies reporting sex, both men and women were included, with a moderate predominance of male participants (mean proportion of males: 63.3 ± 6.7%). No relevant baseline age differences between intervention groups were observed within studies.

**Table 1:** Study Characteristics and Intervention Details.

Study	Study Design	Stroke Phase	Etiology	Sex	Groups (*n*)	Age (Years)	Intervention (MI/MT)	Intervention Volume			Outcomes	Results
								Weeks	Frequency	Duration (Minutes)		
De et al., 2017 [34]	RCT	Subacute	n.i.	n.i.	MI (15)	50–65 years	Kinesthetic MI of ankle DF/EV + CRT	4	5	MI: 30 CRT:30	FMA-LE MAS 10MWT	MI > MT: FMA-LE; No between-group differences: MAS, 10MWT
					MT (15)		Mirror feedback of ankle DF/EV + CRT	4	5	MT: 30 CRT:30		
Finney et al., 2024 [35]	RCT	Acute	Ischemic	F (36.7%) M (63.3%)	MI (15)	n.i.	Functional MI + physical practice	7	3	Total: 60	ARAT (total and subscales)	MI > MT: ARAT total, gross movement; both groups improved
					MT (15)	n.i.	Functional MT of same tasks	7	3	Total: 60		
Jain et al., 2024 [36]	RCT	Acute	Ischemic and hemorrhagic	F (43.3%) M (56.7%)	MI (15)	56.1	Internal visual MI of ankle DF/EV + CRT	4	5	MI: 30 CRT:30	FMA-LE MAS 10MWT	MI > MT: FMA-LE; No between-group differences: MAS, 10MWT
					MT (15)	56.6	Mirror feedback of ankle DF/EV + CRT	4	5	MT: 30 CRT:30		
Lee et al., 2019 [33]	Comparative experimental	Subacute	Ischemic and hemorrhagic	n.i.	MI-Mild (6)	71.6	Audio-guided MI (upper limb)	4	n.i.	MI: 20 Relax: 10	BBT JTHFT MFT	Cognition-dependent effects: MI > MT (mild: JTHFT); MT > MI (severe: BBT, JTHFT, MFT)
					MT-Mild (6)	73.8	Mirror feedback (upper limb)	4	n.i.	n.i.		
					MI-Severe (6)	72.0	Audio-guided MI (upper limb)	4	n.i.	MI: 20 Relax: 10		
					MT-Severe (6)	70.7	Mirror feedback (upper limb)	4	n.i.	n.i.		
Margrett et al., 2019 [38]	RCT	n.i.	n.i.	n.i.	MI (31)	age between 30 and 80 years	Visual and kinesthetic MI of gait-related tasks + CRT	6	5	MI: 15 CRT:30	Step length Stride length Cadence 10MWT	Both MI and MT improved gait outcomes; no between-group differences
					MT (31)		MT during gait-related functional tasks + CRT	6	5	MT: 15 CRT:30		
Thara et al., 2015 [37]	RCT	Subacute	Ischemic	F (30.0%) M (70.0%)	MI (15)	51.9	Task-specific MI (upper limb) + home program	10	3	Total: 60	ARAT (total score and subscales)	MI > MT: ARAT hand function
					MT (15)	52.7	Task -specific MT (upper limb) + home program	10	3	Total: 60		

Abbreviations: 10MWT, 10-Meter Walk Test; ARAT, Action Research Arm Test; BBT, Box and Block Test; CRT, Conventional Rehabilitation Therapy; DF/EV, dorsiflexion/eversion; F, female; FMA-LE, Fugl–Meyer Assessment, lower extremity; JTHFT, Jebsen–Taylor Hand Function Test; M, male; MAS, Modified Ashworth Scale; MFT, Manual Function Test; MI, Motor Imagery; MI-Mild, Motor Imagery in patients with mild cognitive impairment; MI-Severe, Motor Imagery in patients with severe cognitive impairment; MP, Mental Practice; MT, Mirror Therapy; MT-Mild, Mirror Therapy in patients with mild cognitive impairment; MT-Severe, Mirror Therapy in patients with severe cognitive impairment; n.i., not informed; RCT, randomized controlled trial.

#### 3.2.3. Stroke Characteristics

Regarding stroke phase, three studies included participants in the subacute phase [33,34,37], two studies focused on the acute phase [35,36], and one study did not report stroke phase [38]. Stroke etiology was inconsistently described. Two studies included only ischemic stroke patients [35,37], two included both ischemic and hemorrhagic strokes [33,36], and two did not report stroke type [34,38]. Anatomical lesion location was poorly reported overall, with only two studies specifying involvement of the middle cerebral artery territory [34,36]. Lesion laterality was reported in only one study, which identified a predominance of right-hemisphere lesions [33].

#### 3.2.4. Cognitive Status

Cognitive status was heterogeneously assessed and explicitly quantified in only one trial. Lee and Cha evaluated cognition using the Korean version of the Mini-Mental State Examination (MMSE-K), stratifying participants into mild (18–23 points) and severe (0–17 points) cognitive impairment groups, with mean MMSE-K values ranging from approximately 20.5 to 21.5 in the mild group and 15 to 16 in the severe group [33]. In contrast, De et al. and Margrett et al. applied cognitive thresholds as eligibility criteria, including only participants with preserved cognitive function (MMSE > 24 and > 25, respectively) [34,38], thereby excluding individuals with moderate-to-severe cognitive impairment [34,38]. Finney et al. did not report a standardized cognitive assessment but excluded participants with behavioral or attentional disturbances and those with global aphasia accompanied by cognitive impairment [35]. Thara et al. and Jain et al. did not report formal cognitive screening instruments, although Jain et al. excluded participants with neglect or apraxia [36,37].

#### 3.2.5. Baseline Functional Status

Baseline functional status was inconsistently reported. Quantitative baseline motor values were provided only by Jain et al., who reported pre-intervention scores for the Fugl–Meyer Assessment (lower extremity, FMA-LE), the 10-Meter Walk Test (10MWT), and the Modified Ashworth Scale (MAS) [36]. Thara et al. reported baseline upper-limb function using the Action Research Arm Test (ARAT), including pre-intervention mean values for both intervention groups [37]. In Lee and Cha, baseline characterization focused primarily on cognitive status without numerical baseline motor scores [33]. The remaining studies described baseline status indirectly through eligibility criteria related to motor recovery stage, mobility, or spasticity [34,35,38]. 

#### 3.2.6. MI and MT Interventions

MI and MT interventions were delivered using task-oriented protocols with comparable structures, although with variability in delivery format and integration with conventional rehabilitation. MI was implemented as guided mental practice supported by verbal instructions and, in several studies, audiovisual cues such as videos of functional tasks or gait patterns and standardized audio recordings [33,35,37,38]. When specified, MI combined visual and kinesthetic components, and an internal imagery perspective was explicitly reported in two studies [36,38]. In the remaining studies, imagery perspective was not described [33,34,35,37].

MT was applied using a mirror positioned along the participant’s midsagittal plane, with the affected limb hidden behind the mirror and the non-paretic limb visible in front of it, allowing the mirror reflection to be perceived as the affected limb when described [35,36,37]. Both MI and MT targeted either the upper or lower extremity depending on study focus, addressing upper-limb function through grasping and object manipulation tasks [33,35,37] or lower-limb function through ankle movements and gait-related tasks [34,36,38].

Across all studies, MI and MT employed functionally meaningful, task-specific activities rather than isolated analytical movements. Session duration ranged from 30 to 60 min, intervention frequency from three to five sessions per week, and total program duration from 4 to 10 weeks, consistently matched between MI and MT within each study. Importantly, neither MI nor MT was delivered as a standalone intervention; both were systematically combined with CRT or physical practice of the same tasks and supervised by a therapist, which limits the ability to isolate the independent effects of MI or MT [34,35,36,37,38].

### 3.3. Summary of Results

Outcome measures were categorized into four domains: motor function, functional performance, spasticity, and gait-related outcomes. Across the included studies, both MI and MT were generally associated with significant pre–post improvements. However, findings from between-group comparisons were inconsistent, with no clear or systematic superiority of one intervention over the other.

#### 3.3.1. Motor Function

Motor function was the most frequently assessed outcome domain. Upper-limb motor function, evaluated using the ARAT, improved significantly from baseline to post-intervention in both MI and MT groups in studies focusing on upper-extremity recovery [35,37,38]. Between-group comparisons yielded mixed results. In one study, both interventions produced significant improvements without consistent superiority across ARAT subdomains [37]. In contrast, another study reported greater post-intervention gains in specific ARAT components (grip and pinch) and total scores in the MI group compared with MT, while other subdomains showed comparable changes between groups [35].

Lower-limb motor function was assessed using the FMA-LE in studies targeting ankle control and gait [34,36]. Both studies reported significant within-group improvements following MI and MT interventions. When between-group differences were examined using change scores (post–pre), a greater improvement in FMA-LE was observed in the MI group in both trials. However, this finding was limited to impairment-level motor recovery and does not indicate a consistent or generalized superiority of MI over MT across outcome domains.

#### 3.3.2. Functional Performance

Functional performance outcomes reflected task execution and manual dexterity. In one study, upper-limb functional performance was assessed using the BBT and the JTHFT [33]. Both MI and MT groups demonstrated significant functional improvements, with subgroup analyses suggesting that participants with milder cognitive impairment achieved greater gains regardless of intervention type. No consistent between-group superiority was reported. Walking performance, evaluated using the 10MWT, improved significantly in both MI and MT groups across studies focused on gait rehabilitation, with no significant differences between interventions [34,36,38].

#### 3.3.3. Spasticity

Spasticity, assessed using the MAS, was reported in two studies [34,36]. Both MI and MT groups showed reductions in muscle tone from baseline to post-intervention, with no significant differences identified between interventions, suggesting similar effects on spasticity.

#### 3.3.4. Gait-Related Outcomes

Gait-related outcomes included walking speed and spatiotemporal parameters. Walking velocity, measured using the 10MWT, increased significantly in both MI and MT groups across all studies assessing gait [34,36,38]. One study additionally reported improvements in spatiotemporal gait parameters, including step length, stride length, and cadence, with comparable gains observed between MI and MT groups and no statistically significant between-group differences [38].

### 3.4. Assessment of Methodological Quality

Methodological quality assessed with the PEDro scale varied across studies, with total scores ranging from 3 to 8. One study achieved a score of 8 [37], two studies scored 7 [35,36], one study scored 6 [33], one scored 5 [34], and one study showed low methodological quality with a score of 3 [38]. Most studies met criteria related to eligibility specification, random allocation, baseline comparability, between-group statistical comparisons, and reporting of point estimates and variability. In contrast, concealed allocation and blinding of participants and therapists were not reported, while assessor blinding was inconsistently addressed.

Overall, the included studies demonstrated low to moderate methodological quality, with recurrent limitations related to allocation concealment and blinding. Detailed PEDro scores for each study are presented in Table 2.

### 3.5. Risk of Bias of the Included Studies

**Figure 2:**
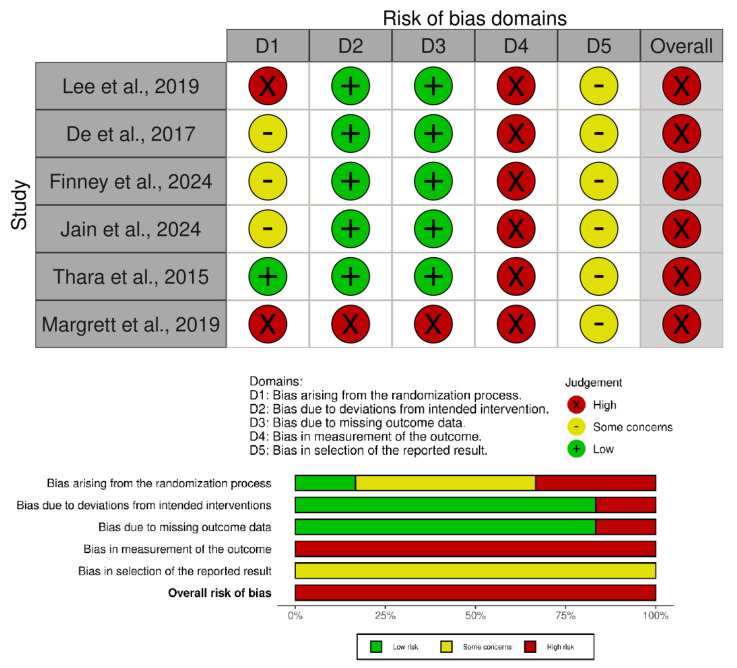
Risk-of-bias summary of the included studies [33,34,35,36,37,38].

Risk of bias was assessed using the Cochrane Risk of Bias 2 (RoB 2) tool, with overall judgments presented in Figure 2. All included studies were judged to be at high overall risk of bias. Inter-rater agreement was high (Cohen’s κ = 0.79), indicating substantial consistency between reviewers. The main sources of bias were related to deviations from intended interventions and outcome measurement, primarily due to the lack of blinding of participants, therapists, and outcome assessors—an inherent limitation of non-pharmacological interventions.

Concerns regarding the randomization process were also frequent, with several studies rated as Some concerns or High risk because of insufficient reporting of sequence generation and allocation concealment. In contrast, bias due to missing outcome data was generally rated as Low risk, indicating acceptable follow-up rates. Bias in the selection of reported results was consistently rated as Some concerns, mainly due to the absence of pre-registered protocols or clearly defined analysis plans.

Overall, although the included studies addressed clinically relevant questions, methodological limitations—particularly related to blinding and deviations from intended interventions—substantially increased the risk of bias and should be considered when interpreting the findings of this review.

## 4. Discussion

This systematic comparative review indicates that both MI and MT are associated with significant pre–post improvements in motor function, functional performance, spasticity, and gait-related outcomes after stroke when applied as adjuncts to CRT. Across the included trials, between-group comparisons showed heterogeneous findings, with no consistent evidence supporting the superiority of either intervention.

Improvements were observed in both upper- and lower-limb outcomes regardless of stroke phase. Although isolated advantages of MI were reported for specific upper-limb subdomains, such as grip and pinch, these effects were not consistently replicated across studies or outcome measures. Overall, the findings suggest that MI and MT provide comparable clinical benefits, likely mediated by shared mechanisms related to task-oriented practice and motor network activation rather than by intervention-specific effects.

The findings of this review are broadly consistent with previous literature examining MI and MT as adjuncts to CRT, while adding novel insight through direct comparison between both interventions. Prior systematic reviews and meta-analyses have suggested that MI may improve motor outcomes after stroke [12,13,14,15]; however, these effects have been characterized by substantial heterogeneity and sensitivity to methodological quality and publication bias. When more rigorous analytical approaches are applied, including formal assessment and correction for publication bias, the magnitude and robustness of MI-related benefits appear reduced and inconsistent [16,17].

In contrast, MT has shown more stable and apparently consistent effects across several systematic reviews, particularly for upper-limb motor recovery and, to a lesser extent, for balance and gait-related outcomes [19,20,21,22,23,24]. However, it is important to note that none of the available meta-analyses on MT have formally evaluated publication bias. Consequently, the apparent consistency of MT effects should be interpreted with caution, as the possibility of selective reporting or small-study effects cannot be excluded.

Within this context, the present review indicates that MT does not confer a systematic advantage over MI when both interventions are directly compared under similar rehabilitation conditions. The benefits attributed to either approach may largely reflect shared, non-specific therapeutic factors—such as task-oriented practice, repetition, therapist supervision, and concurrent conventional rehabilitation—rather than modality-specific mechanisms unique to MI or mirror-induced visual feedback.

Across outcome domains, MI and MT demonstrated largely comparable effects, with only domain-specific and modest differences. For upper-limb motor function, both interventions produced significant improvements, while isolated advantages of MI were reported in specific studies and limited subdomains, such as grip and pinch strength [35,37]. Importantly, these findings were study-specific and were not consistently replicated across trials or outcome measures, suggesting context-dependent effects rather than robust or generalizable superiority.

Lower-limb motor function and gait-related outcomes showed largely comparable effects between interventions, despite a study-specific advantage of MI for impairment-level motor recovery (FMA-LE) based on change-score analyses [36,38]. Similarly, functional performance measures and reductions in spasticity improved to a similar extent in both groups [33,36]. Overall, these findings indicate that MI and MT exert largely overlapping effects on motor and functional recovery, with limited evidence supporting intervention-specific advantages beyond selected upper-limb tasks. The feasibility of a quantitative synthesis was carefully considered; however, meta-analysis was not deemed appropriate due to the limited number of directly comparable studies per outcome and the heterogeneity in outcome selection and reporting. Although some measures were shared across trials—FMA-LE, ARAT, 10MWT, and MAS—these outcomes were generally reported in only two studies and often with insufficient statistical detail or inconsistent formats. Future studies should prioritize the use of harmonized outcome measures and standardized reporting to enable robust quantitative synthesis and future meta-analyses directly comparing MI and MT.

Cognitive status emerged as a potentially relevant factor influencing responsiveness to MI and MT. Among the included studies, only Lee and Cha [33] explicitly stratified participants according to cognitive impairment, providing direct comparative evidence across cognitive levels. In that study, MI was associated with greater functional gains in participants with milder cognitive impairment, whereas MT appeared more effective in those with more severe cognitive deficits. Although cognitive status was not treated as a primary analytical axis in the present review, the stratified findings reported by Lee and Cha suggest that cognition may play a central role in modulating responsiveness to MI versus MT [33]. The fact that this signal emerged from a single study underscores both its potential clinical relevance and the current limitations of the evidence base. Future trials should therefore be specifically designed to incorporate cognitive status as a core stratification or analytical factor, using standardized cognitive assessments and adequately powered subgroup analyses, to determine whether cognition-dependent differential effects between MI and MT can be confirmed and generalized. This pattern further suggests that the successful application of MI may depend on preserved cognitive resources, including attention, working memory, and the ability to generate and sustain internal motor representations [11].

Beyond behavioral cognitive measures, emerging neurophysiological evidence from the broader literature suggests that inter-individual variability in responsiveness to MI may be partly explained by differences in the functional integrity of sensorimotor networks [11,39]. Resting-state EEG features, particularly within the mu and beta bands, have been proposed as potential markers of MI ability and motor simulation capacity [11,39,40]. Individuals exhibiting more clearly defined sensorimotor rhythms at rest tend to show larger and more consistent event-related desynchronization during MI, which has been associated with more effective motor simulation [41]. This perspective aligns with the distinction between responders and non-responders described in MI-based brain–computer interface research (BCI literacy) [39,42]. From a clinical standpoint, simple EEG-derived markers obtained at rest may help identify patients more likely to benefit from MI-based interventions; however, evidence remains limited and further research is needed to clarify their clinical applicability [39,40]. These considerations should be interpreted as theoretical perspectives and potential directions for future research, as neurophysiological measures were not directly assessed in the studies included in this review.

Taken together, these observations suggest that cognitive status should not be considered in isolation, but rather as part of a broader neurocognitive profile influencing treatment response. Integrating behavioral cognitive assessment with basic neurophysiological markers may represent a feasible and clinically relevant approach to guide the indication of MI versus MT in stroke rehabilitation. However, this hypothesis remains largely unexplored in clinical trials and warrants systematic investigation in future studies specifically designed to examine cognition–neurophysiology–intervention interactions.

From a clinical standpoint, these findings support the interpretation of MI and MT as complementary rather than mutually exclusive interventions. Given the lack of consistent superiority of one approach over the other, clinical decision-making should prioritize patient characteristics and rehabilitation goals. MT may be particularly suitable for patients with greater cognitive impairment, reduced imagery capacity, or in earlier stages of recovery, where externally driven visual feedback can facilitate task engagement. In contrast, MI may be better suited for individuals with preserved cognitive function and imagery ability, especially when rehabilitation targets fine motor control or task-specific motor refinement.

Several limitations should be considered when interpreting the findings of this review. From a methodological perspective, all included studies were characterized by low-to-moderate PEDro scores and were classified as having a high overall risk of bias according to the RoB 2 tool, mainly due to lack of blinding, deviations from intended interventions, and selective outcome reporting. Importantly, this consistently high risk of bias substantially reduces confidence not only in the magnitude of the observed effects, but also in the validity of the between-group comparisons between MI and MT. As a result, the absence of consistent superiority of either intervention should be interpreted with caution, as it may partly reflect methodological constraints rather than true equivalence. These sources of bias may also contribute to the heterogeneity and inconsistency of comparative findings across studies.

From a clinical perspective, the included trials were generally based on small sample sizes and showed substantial heterogeneity in stroke phase, targeted limb (upper vs. lower), intervention content, and intervention duration, which limits generalizability and complicates direct comparisons across studies. Moreover, MI and MT were almost exclusively applied as adjuncts to conventional rehabilitation therapy or physical practice, making it difficult to isolate the specific contribution of each intervention and restricting causal attribution to modality-specific effects. To our knowledge, only a very limited number of studies have evaluated MI as a standalone intervention without concomitant CRT or physical practice, with only one such study identified within the scope of this review [43]. From an analytical perspective, heterogeneity in outcome selection and reporting, together with the limited number of comparable studies per outcome, precluded the performance of a quantitative synthesis. Although some outcomes—such as Fugl–Meyer, ARAT, 10MWT, and MAS—were shared across studies, these were typically reported in only two trials and often with insufficient statistical detail or inconsistent formats. The lack of harmonized outcome measures and standardized reporting further limited the feasibility of meta-analysis and highlights the need for greater methodological consistency in future research.

Future research should prioritize adequately powered RCTs with improved methodological rigor, including better control of bias and transparent reporting. In line with recent meta-analytical evidence, systematic assessment and correction of publication bias are essential, as unadjusted pooled effects may substantially overestimate the benefits of MI and related interventions. Further efforts should focus on clearer standardization of MI protocols and on direct comparisons between MI, MT, and their combined application to determine whether effects extend beyond those attributable to CRT alone.

In summary, MI and MT should not be regarded as competing interventions, but as complementary strategies addressing partially overlapping patient needs. Current evidence does not justify the generalized preference of one approach over the other, nor their routine implementation without consideration of methodological quality and bias. Progress in this field is therefore likely to depend on improved trial design, rigorous bias control, and the integration of cognitive and neurofunctional profiling to better delineate the contexts in which these interventions may offer clinically meaningful benefit.

## 5. Conclusions

Current evidence indicates that MI and MT produce largely comparable improvements in motor and functional outcomes after stroke when applied as adjuncts to CRT, with no consistent or robust evidence supporting the superiority of one intervention over the other. Reported differences between approaches are generally modest, domain-specific, and inconsistently replicated across studies. Importantly, the absence of consistent between-group differences should be interpreted in light of limited statistical power, methodological heterogeneity, and high risk of bias across studies, and does not necessarily indicate true equivalence between interventions.

Interpretation of these findings is constrained by small sample sizes, inter-study heterogeneity, and the overall moderate methodological quality and high risk of bias of the included trials, as well as by the frequent integration of both interventions within multimodal rehabilitation programs, which limits causal attribution to intervention-specific effects. Within these constraints, MI and MT should be regarded as clinically comparable and complementary strategies rather than competing approaches.

From a clinical perspective, intervention selection should therefore be guided by individual patient characteristics—particularly cognitive status and imagery ability—rather than assumptions of inherent efficacy differences. Future progress in this field will depend on methodologically robust trials with adequate statistical power, harmonized outcome measures, standardized intervention protocols, improved control of bias, and systematic assessment of publication bias, alongside greater integration of cognitive and neurofunctional profiling to support personalized rehabilitation strategies.

## Figures and Tables

**Table 2:** PEDro scale methodological quality of the included studies.

Study	1	2	3	4	5	6	7	8	9	10	11	Total
Lee et al., 2019 [33]	Y	N	N	Y	N	N	N	Y	Y	Y	Y	6
De et al., 2017 [34]	Y	Y	N	N	N	N	N	Y	Y	Y	N	5
Finney et al., 2024 [35]	Y	Y	N	Y	N	N	N	Y	Y	Y	Y	7
Jain et al., 2024 [36]	Y	Y	N	Y	N	N	N	Y	Y	Y	Y	7
Thara et al., 2015 [37]	Y	Y	Y	Y	N	N	N	Y	Y	Y	Y	8
Margrett et al., 2019 [38]	Y	N	N	N	N	N	N	N	N	Y	Y	3
Note: Y; Yes; N, No.												

## Data Availability

No new data were generated in this study. All data supporting the findings of this systematic review are derived from published clinical trials and are available within the article and its Supplementary Materials.

## Supplementary Materials

The following supporting information can be downloaded at: https://www.mdpi.com/article/10.3390/life16020306/s1. Table S1: Search database.

## Abbreviations

The following abbreviations are used in this manuscript:

ARAT	Action Research Arm Test
BBT	Box and Block Test
BCI	Brain–Computer Interface
CRT	Conventional Rehabilitation Therapy
EEG	Electroencephalography
ERD	Event-Related Desynchronization
FMA-LE	Fugl–Meyer Assessment – Lower Extremity
JTHFT	Jebsen–Taylor Hand Function Test
MAS	Modified Ashworth Scale
MI	Motor Imagery
MMSE	Mini-Mental State Examination
MMSE-K	Korean Version of the Mini-Mental State Examination
MT	Mirror Therapy
PEDro	Physiotherapy Evidence Database
PRISMA	Preferred Reporting Items for Systematic Reviews and Meta-Analyses
PROSPERO	International Prospective Register of Systematic Reviews
RCT	Randomized Controlled Trial
RoB 2	Risk of Bias tool, version 2
10MWT	10-Meter Walk Test
